# Induced pluripotent stem cell for modeling Pompe disease

**DOI:** 10.3389/fcvm.2022.1061384

**Published:** 2022-12-22

**Authors:** Wenjun Huang, Yanmin Zhang, Rui Zhou

**Affiliations:** ^1^National Regional Children's Medical Center (Northwest), Key Laboratory of Precision Medicine to Pediatric Diseases of Shaanxi Province, Xi'an Key Laboratory of Children's Health and Diseases, Shaanxi Institute for Pediatric Diseases, Xi'an Children's Hospital, Affiliated Children's Hospital of Xi'an Jiaotong University, Xi'an, China; ^2^Department of Cardiology, Xi'an Children's Hospital, Affiliated Children's Hospital of Xi'an Jiaotong University, Xi'an, China

**Keywords:** Pompe disease, induced pluripotent stem cell, glycogen storage disease type II, GAA, disease modeling

## Abstract

Pompe disease (PD) is a rare, autosomal recessive, inherited, and progressive metabolic disorder caused by α-glucosidase defect in lysosomes, resulting in abnormal glycogen accumulation. Patients with PD characteristically have multisystem pathological disorders, particularly hypertrophic cardiomyopathy, muscle weakness, and hepatomegaly. Although the pathogenesis and clinical outcomes of PD are well-established, disease-modeling ability, mechanism elucidation, and drug development targeting PD have been substantially limited by the unavailable PD-relevant cell models. This obstacle has been overcome with the help of induced pluripotent stem cell (iPSC) reprogramming technology, thus providing a powerful tool for cell replacement therapy, disease modeling, drug screening, and drug toxicity assessment. This review focused on the exciting achievement of PD disease modeling and mechanism exploration using iPSC.

## Introduction

As a lysosomal glycogen accumulation disease, Pompe disease (PD) is an autosomal recessive disorder caused by the mutation of the *GAA* gene encoding α-glucosidase (AαGlu), leading to glycogen over-accumulation in the lysosomes of different tissues, especially in the skeletal and cardiac muscles (1, 2). A huge amount of lysosomal glycogen accumulation in the skeletal muscles and cardiomyocytes eventually leads to death from cardiorespiratory failure (3). PD can be classified into two types based on the onset age of the disease, infantile-onset Pompe disease (IOPD) and late-onset forms (LOPDs). For different patients, the tissue injury and clinical symptoms depend on the varied residual enzyme activity, and thus, this determines the prognosis of patients with PD. Patients with IOPD with absent AαGlu activity usually manifest progressive skeletal muscle weakness and cardiac hypertrophy and die within a few months after birth without effective treatment as a result of heart and respiratory failure (4). Patients with LOPD often develop reduced AαGlu activity and have a slower skeletal muscle weakness progression, rarely showing cardiac muscle involvement (5, 6).

Pompe disease animal models have made a significant contribution toward exploring pathogenesis. Several PD mouse models have been used to study PD's pathophysiological characteristics (7, 8), including the application of gene therapy (9). Recently, the technology of induced pluripotent stem cell (iPSC) reprogramming dramatically accelerated PD research advancement. The iPSC generated from patients with PD (PD-iPSC) can be successfully differentiated into various somatic cells, such as cardiomyocytes and the skeletal muscles, in which the phenotypes and pathological features with the same genetic background can be recapitulated *in vitro*. PD-iPSC modeling can be a good tool to probe the pathogenic mechanism and new valuable therapeutic strategies. This review aimed to make an overview of PD-iPSC modeling, including disease pathogenesis, different target models derived from iPSC, and a summary of research progress about PD.

## GAA function and PD pathogenesis

Alpha-glucosidase (GAA) enzyme deficiency caused glycogen accumulation within the swollen lysosomes and probable ruptured lysosomes, thus manifesting as a multisystem disorder, especially in the skeletal and cardiac muscles (10).

The *GAA* gene is approximately 18.3 kb long and localized on chromosome 17q25.3 (11) with a 2,859 bp-length cDNA encoding the protein enzyme, AαGlu, with 952 amino acids (2), which hydrolyses lysosomal glycogen to glucose and then preventing glycogen storage in lysosomes (12). GAA experiences the sequential processes of protein modification in different organelles, from glycosylation in the endoplasmic reticulum to mannose 6-phosphate addition in the Golgi (13) and finally to enzyme digestion in the lysosome where the 110 kDa precursor is converted into d 76- and 69-kDa mature forms with enhanced enzyme activities (14).

So far, 2,075 *GAA* mutations consisting of 1,205 in exons and 870 in introns have been identified, suggesting the highly heterogeneous spectrum of *GAA* mutations (15–18), which lead to varying expression levels and/or GAA protein activity. Different *GAA* mutations may partially explain different expressions and GAA protein activity. Point mutations can influence posttranscriptional splicing or directly change the GAA protein function, while deletions and/or insertions of DNA fragments may yield unstable mRNA transcription, thus finally affecting protein translation, posttranslational modifications, trafficking into the lysosome, and glycogenolysis activity of GAA. As the most reported mutation type, missense mutations of the *GAA* gene occurring in the unexposed amino acid residues often result in misfolding of the 3D protein structure (19, 20).

## Pompe disease iPSC modeling

Pompe disease animal models have made a significant contribution toward promoting PD research. The murine PD models exhibit cellular and tissular phenotypes similar to those in human beings, such as decreased GAA activity, obvious lysosome glycogen accumulation, and abnormal ultrastructure in the lysosome, but their integral clinical feature differs greatly from patients (21). In contrast, iPSC-derived target cells could recapitulate the characteristic phenotype in the *in vitro* dish, which is equivalent to the iPSC donor. Therefore, iPSC derived from patients with a hereditary disease can provide an ideal cell and/or organoid resource for disease modeling and mechanism study (22–24). Last decade, more than ten studies utilized PD-iPSC modeling to explore the phenotypes and pathogenetic mechanism, which greatly expand the understanding of the disease (Table 1).

**Table 1:** Characteristics of Pompe disease iPSC models.

**Disease type**	**Reprogram** **-ming methods**	**Target cells**	**Main research method**	**Main observations**	**Potential durgs/** **therapeutics**	**Mutations**	**References**
GAA-KO mouse	Retrovirus	Skeletal myocytes	Electron microscopic Enzyme activity assay PAS/ACP staining	Morphological features GAA enzyme activity Glycogen accumulation	(–)	Not mentioned	(31)
IOPD	Retrovirus	Cardiomyocytes	Cellular glycogen content Enzyme activity assay Measurement of intact cellular respiration Electron microscopy Microarray analysis	Glycogen accumulation GAA enzyme activity Ultrastructural aberrances Metabolomics changes	rhGAA L-carnitine	c.1935C>A c.1935C>A/c.2040+1G>T	(25)
IOPD LOPD	Retrovirus	(–)	PAS staining Electron microscopy	Glycogen accumulation Ultrastructural aberrances	rhGAA	Not mentioned	(22)
IOPD	Lentivirus	Cardiomyocytes	Electron microscopy Enzyme activity assay Engineered cardiac tissue preparation and functional Testing Isoelectric focusing studies N-Linked glycan identification by MALDI-TOF-MS	Glycogen accumulation GAA enzyme activity Contractile function Autophagic dysfunction Golgi-based glycosylation	(–)	c.2532-2673del c.1441delT/2237G>A	(26)
LOPD	(–) Pre-established	Cardiomyocytes	Glycogen content analysis Electron microscopy Enzyme activity assay	Glycogen accumulation GAA enzyme activity Lysosomal enlargement	Gene therapy (GAA)	c.796C>T/c.1316T>A	(27)
Not mentioned	(–) Pre-established	Skeletal myocytes	Glycogen content analysis Enzyme activity assay Electron microscopy	Glycogen accumulation GAA enzyme activity Ultrastructural aberrances	Gene therapy (GAA and TFEB)	Not mentioned	(32)
LOPD PD mouse	(–) Pre-established		CE-MS Analysis Glutathione redox ratio assay Reactive oxygen species assay	Metabolomic profiling Oxidative stress-associated metabolic parameters	(–)	Not mentioned	(28)
IOPD	Sendai virus	Skeletal myocytes	PAS staining Glycogen analysis Electron microscopy mTORC1 activation assay Rapamycin analysis Metabolomic analysis Gene expression profiling and microarray data analysis	Lysosomal glycogen accumulation GAA enzyme activity mTORC1-related signaling Energy metabolism Mitochondrial oxidative function	rhGAA	c.1880C>T c.796 C>T/c.1316 T>A. c.1798C>T/c.2481+1 G>A	(33)
IOPD	Sendai virus	Hepatocytes	PAS staining Glycogen analysis Immunofluorescence and electron microscopy	Lysosomal glycogen accumulation rhGAA rescue for lysosomal glycogen accumulation	rhGAA	c.1880C > T c.796 >T/c.1316T>A c. 1798C>T/c.2481+1 G>A	(39)
IOPD	Lentivirus	Skeletal myocytes	Enzyme activity assay Electron microscopy DIC analysis	GAA enzyme activity Ultrastructure Contraction dynamics	(–)	c.1441delT/2237G>A	(34)
IOPD	Artificially induced	Skeletal muscle cell (Ai-SKMC)	The DeepNEU simulations DeepNEU platform specification	The aiPSC simulations The transdifferentiated skeletal muscle cell simulation (AI-SkMC) of IOPD Application of the validated aiSkMC simulation to disease modeling, biomarker identification and drug discovery	(–)	Not mentioned	(36)
IOPD	Sendai virus	(–)	Giemsa-banding Immunofluorescence and flow cytometry	Karyotype of the iPSC line Pluripotent markers Trilineage differentiation potential	(–)	c.1822C > T, p.R608X + c.2662G > T, p.E888X	(30)
IOPD	Retrovirus	Neural cells	Cellular glycogen content Enzyme activity assay PAS staining	Glycogen accumulation GAA Enzyme activity Neural cells apoptosis	Ebselen, Wortmannin, and PX-866	Patient 1: 1935 C>A; Patient 2: 1935 C>A/2040+1G>T	(40)
IOPD	Sendai virus	Neural Stem Cells	Cellular glycogen content Enzyme activity assay	Glycogen accumulation GAA Enzyme activity	Hydroxypropyl- β-cyclodextrin and δ-ocopherol	c.2560C > T	(41)

IOPD, infantile-onset Pompe disease; LOPD, late-onset Pompe disease.

### Glycogen accumulation in iPSC

Glycogen accumulation in PD-iPSC was investigated in the early stages. Higuchi et al., successfully established iPSC from patients with IOPD and LOPD. They observed massive glycogen granules in IOPD- and LOPD-iPSCs, but the IOPD-iPSCs exhibited more glycogen accumulation compared with LOPD-iPSCs. In addition, treatment with recombinant human lysosomal alpha-glucosidase (rhGAA) could significantly alleviate glycogen particle accumulation in the lysosomes of IOPD-iPSCs dose-dependently (22). This study revealed that glycogen accumulation, a hallmark of PD pathophysiological phenotypes, could occur as early as the iPSC stage.

### Pompe disease iPSC-derived cardiomyocytes

Huang et al. (25) established iPSCs from two patients with late-onset Pompe disease (LOPD-iPSCs) carrying *GAA* mutations and derived cardiomyocytes (PD-iCM) from PD-iPSC. It was shown that a mass of glycogen accumulated in PD-iCM, underlying the ultrastructural aberrances including swollen mitochondria, the formation of vacuoles containing glycogen particles, and the formation of autophagosome-like structures. The above major pathologic phenotypes of PD-iPSC-derived cardiomyocytes were alleviated by rhGAA (25).

Raval et al. (26) reprogrammed IOPD skin fibroblasts into iPSC cells and differentiated them into cardiomyocytes. They found that, in the PD-iPSC-derived cardiomyocytes, GAA activity was undetectable and lysosomes filled with pathognomonic glycogen were observed. Contractile properties and autophagy of PD-iPSC-derived cardiomyocytes were not impaired, exhibiting the comparable feature of the control group. It was explained by the authors that contractile dysfunction may not be the major stimulus of hypertrophic cardiomyopathy secondary to the PD, and autophagic dysfunction is not central to early Pompe cardiomyopathy in humans. However, several factors including the culture system, the experimental condition, and the detection timepoint may also influence the results. Nevertheless, they found that PD-iPSC-derived cardiomyocytes produced lysosome-associated membrane proteins (LAMPs) lacking appropriate glycosylation, resulting from the loss of the lysosomal glycogen hydrolyzing ability (26). Glycan processing abnormality due to glycosylation deficiency in lysosomes may contribute to the pathophysiology of Pompe cardiomyopathy.

In another set of experiments, Sato et al. (27) discovered that glycogen accumulation and lysosome enlargement could also be observed in LOPD-iPSCs and LOPD-iPSC-CMs. Especially, they corrected the defect by *GAA* gene overexpression using the lentiviral vector, resulting in alleviated glycogen accumulation and enhanced AαGlu activity (27). Furthermore, they concluded that dysfunctional mitochondria and aggravating oxidative stress are likely involved in cardiac complications caused by the PD after performing the metabolomic assay of PD-iPSC-derived cardiomyocytes cells. It was further confirmed using the genetic engineering mouse PD model, suggesting that oxidative stress and an impaired mitochondrial function may underlie the pathogenesis of late-onset PD (28).

Although patients with IOPD frequently manifest hypertrophic cardiomyopathy, the mechanism of hypertrophic cardiomyopathy caused by the loss of GAA activity remains to be clarified. Our team has been focusing on PD disease for the last 5 years. We previously reported four IOPD cases carrying four complex *GAA* gene mutations (29). Additionally, we also reprogrammed peripheral blood mononuclear cells (PBMC) from one of the patients with IOPD to generate induced pluripotent stem cells (IOPD-iPSCs) carrying compound mutations of the *GAA* gene (R608X and E888X) (30). Together with cardiomyocytes' differentiation from iPSCs, the study provided another ideal *in vitro* cardiac hypertrophy model based on the IOPD-iPSCs.

### Pompe disease iPSC-derived skeletal muscle

Having generated iPSC from a mouse model with PD, Kawagoe et al. (31) successfully differentiated skeletal muscle cells from mouse PD-iPSCs. It was shown that the derived skeletal muscle cells exhibited massive glycogen accumulation in lysosomes (31). These results indicate that the iPSC-derived skeletal muscle cells generated from a murine model could also be a useful disease model for pathogenesis investigation and skeletal muscle treatment in PD. Using skeletal muscle cells from PD patient-specific iPSC, Sato et al. (32) found that lentivirus-delivered GAA remarkably decreased the number of glycogen granules *via* increased GAA enzyme activity. In addition, the therapeutic effect of GAA overexpression could be further improved by introducing transcription factor EB (TFEB), a transcription factor regulating biogenesis and lysosome autophagy (32). Yoshida et al. (33) generated a skeletal muscle model of IOPD with patient-specific iPSCs. The accumulation of lysosomal glycogen was clear and was rescued in a dose-dependent manner by rhGAA. They further demonstrated that the signaling pathway mediated by the mammalian/mechanistic target of rapamycin complex 1 (mTORC1) was inhibited in myocytes derived from IOPD-iPSCs, implying that disturbed mTORC1 signaling may participate in the pathogenesis of skeletal muscle damage in IOPD (33). Recent advances in bioengineering provide multifactorial and multidimensional cell culture strategies that more closely mimic the native biological microenvironment. Based on the micropatterned technology, Jiwlawat et al. successfully generated regularly aligned skeletal muscle cells, which spontaneously contract specifically along the long axis of the myotube. More importantly, the phenotype of aberrant accumulation of lysosomal glycogen particles was more clearly observed (34). Esmail and Danter utilized computer simulation and artificial intelligence (AI) learning to generate computer-simulated induced pluripotent stem cells (AI-iPSCs) and differentiated skeletal muscle cells (AI-iSkMCs) to assist IOPD research and drug screening. Calcium disorder and mitochondrial dysfunction were accurately predicted in IOPD-AI-iSkMC. Furthermore, the L-type calcium channel (LTCC) was precisely identified as a biomarker using IOPD-AI-iSkMC simulation, which has been previously proven to be upregulated in the muscle cells from the mouse and human PD models (35). This suggests a huge potential for computational simulation and artificial intelligence technologies to be used in future iPSC-based research for disease modeling and biomarker discovery (36). It should be noted that there remains room for the improvement of the artificially induced pluripotent stem cells. The lack of complete data of rare disease, including PD, and of complete understanding and knowledge regarding stem cells is a major obstacle that prevents further optimized iPSC simulation.

Recently, several strategies (37, 38) have been developed to enhance the therapeutic effect of enzyme replacement therapy in the primary human skeletal muscle cells, such as the moss-GAA strategy, by which the muscle cells could have better recombinant GAA protein uptake with decreased posttranscriptional modification (37) and by targeting antisense oligonucleotides (AONs) which could help to correct aberrant splicing and restore the reading frame, thus increasing the ratio of GAA protein with normal function (39). It is worth looking forward to the application of these strategies in PD patient-derived iPSC models.

### Pompe disease iPSC-derived hepatocytes

In addition to muscle tissues, the liver is another frequently involved organ in patients with IOPD. Using IOPD patient-iPSC generated, Yoshida et al. (39) successfully constructed an *in vitro* PD liver model, evidenced by aberrant glycogen accumulation in lysosomes and dose-dependent ameliorated glycogen accumulation by rhGAA treatment. It may provide a potent PD liver cells model for drug screening (39).

### Pompe disease iPSC-derived neural cells

The central nervous system is another organ involved in infant-onset PD. In *in vitro* terminally differentiated neural cells (40) and neural stem cells (41) derived from IOPD-iPSCs, PD-related phenotype, including abnormal glycogen accumulation and sharply decreased GAA activities, was recapitulated. More importantly, they also demonstrated potential as a drug-screening model. Using the IOPD-iPSC-derived neurons, Huang et al. (40) successfully screened three potential compound candidates for PD treatment, Ebselen (antioxidant), Wortmannin (GSK3 activator), and PX-866 (PI-3K inhibitor). All these small molecules could increase the GAA activity of Pom-iPSC-derived neurons. In the IOPD-iPSC-derived neural stem cells, Cheng et al. (41) found that hydroxypropyl-β-cyclodextrin (cyclic oligosaccharide) and δ-tocopherol (a vitamin E component) could synergize and amplify the treatment efficiency of rhGAA on the IOPD-iPSC-derived neural stem cells, evidenced by the alleviated autophagy and lipid droplet accumulation. It suggests that IOPD-iPSC-derived neural cells serve as a promising *in vitro* drug screening model for drug discovery in the context of correcting central nervous system defects.

## Expectation

**Figure 1:**
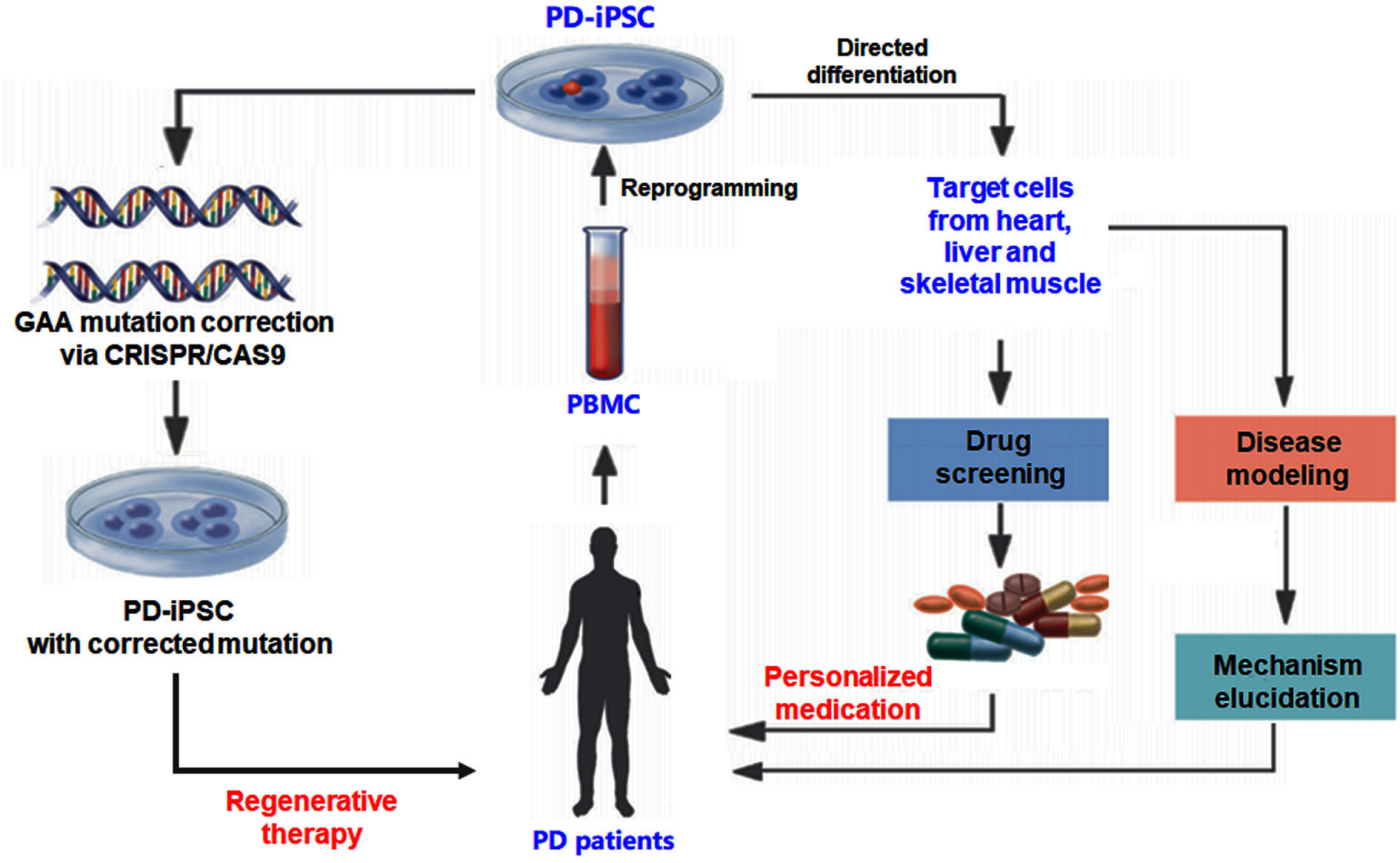
Experimental strategy using human iPSC for Pompe disease (PD) study. The iPSC generated from patients with PD can be differentiated into different cell types to investigate the pathogenesis and develop drug screening for individualized treatment. Gene-editing strategies such as CRISPR-CAS9 can be used to correct disease-causing mutations to achieve gene therapy.

As summarized in Figure 1, the rapid advances in iPSC technology allow the researchers to generate various PD target cells by reprogramming after acquiring the specimen from patients with PD. The disease-specific target cells, including cardiomyocytes and skeletal myocytes, have the patients' genetic information (42, 43). The target cells derived from iPSCs also provide an ideal model to investigate the pathogenesis and develop drug screening for individualized treatment. Making gene therapy in *ex vivo* using a gene-editing strategy possible is another encouraging progress for the iPSC-based PD study. Target cells derived from iPSCs also act as an ideal model to carry out drug screening for personalized treatment (44). This makes *ex vivo* gene therapy by gene-editing strategy a possibility (Figure 1). Gene-editing strategies can be used for the correction of disease-causing mutations to achieve gene therapy. The rapid development of the 3D culture system and new biomaterials give opportunities for the growth of the organoid disease model for PD, which could bridge the gap between *in vitro* cell research and *in vivo* animal models (44). The iPSC-derived target cell model and the organoid disease model are regarded as valuable tools to further drug discovery.

## Author contributions

Literature research and manuscript writing were performed by WH. Manuscript editing was done by RZ. Manuscript revision/review was carried out by RZ and YZ. All authors read and approved the final manuscript.
